# Supplementary material for: ZMYM2 is essential for methylation of germline genes and active transposons in embryonic development
Source: Nucleic Acids Res. 2023 Jul 3;51(14):7314–29. doi: 10.1093/nar/gkad540 (PMC10415128; doi:10.1093/nar/gkad540)
Supplement: gkad540_Supplemental_Files [file gkad540_supplemental_files.zip › 230622 Combined Supplementary Figures.pdf]

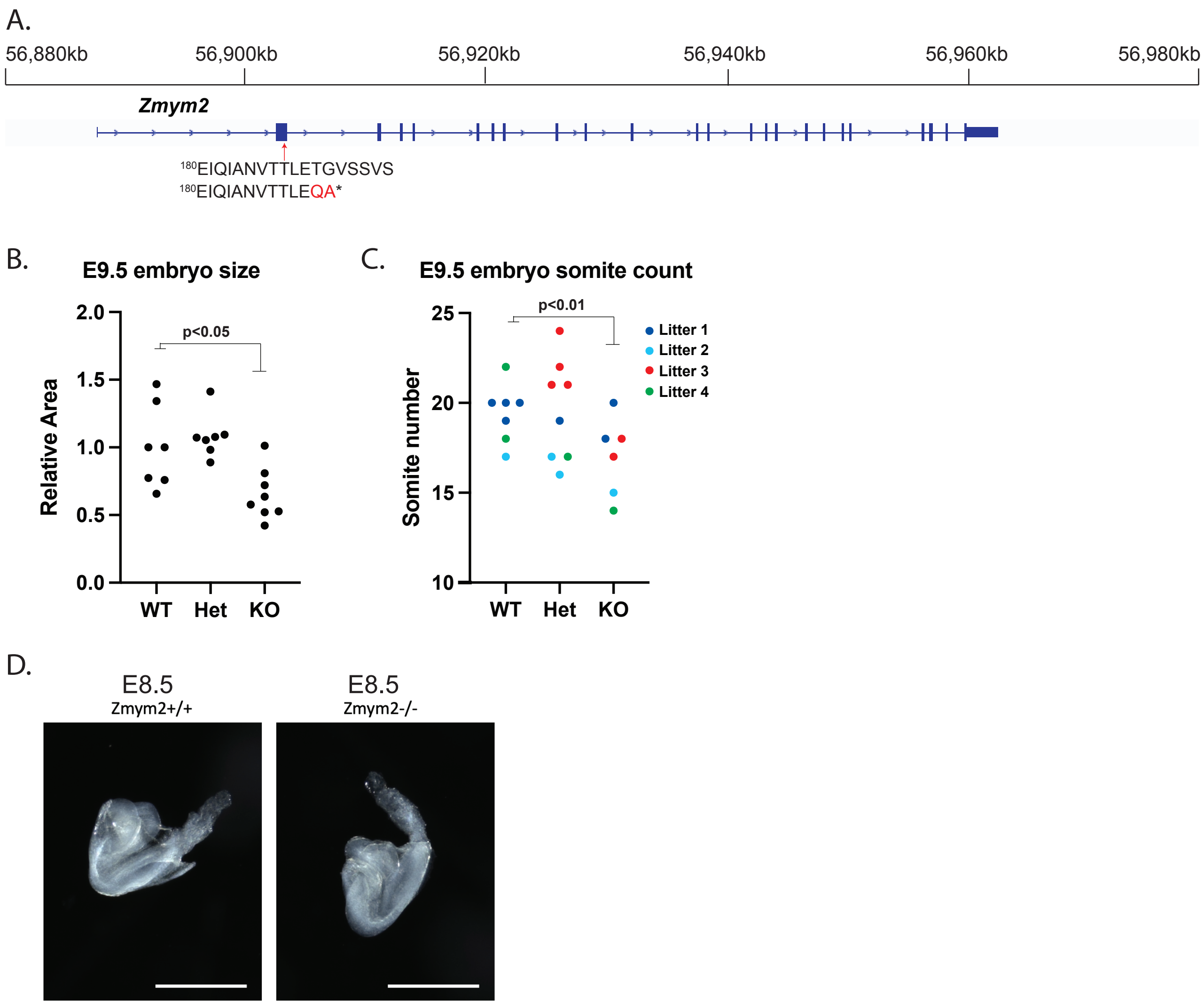

Supplementary Figure S1. Reduced size and developmental delays in *Zmym2*$^{-/-}$ embryos. (**A**) Schematic representation of the nonsense point mutation in *Zmym2-* allele. (**B**) Relative size of embryos measured as 2D area in embryos in sagittal position normalized by litter as a ratio to average *Zmym2*$^{+/+}$ area. p-value calculated on basis of one-tailed t-test. (**C**) Somite counts of E9.5 embryos per genotype. Each litter is represented in a different color to account for litter variability. Note that *Zmym2*$^{-/-}$ mice show reduced somite count within each litter. p-value calculated on basis of one-tailed t-test. (**D**) Images of E8.5 *Zmym2*$^{+/+}$ and *Zmym2*$^{-/-}$ embryos.

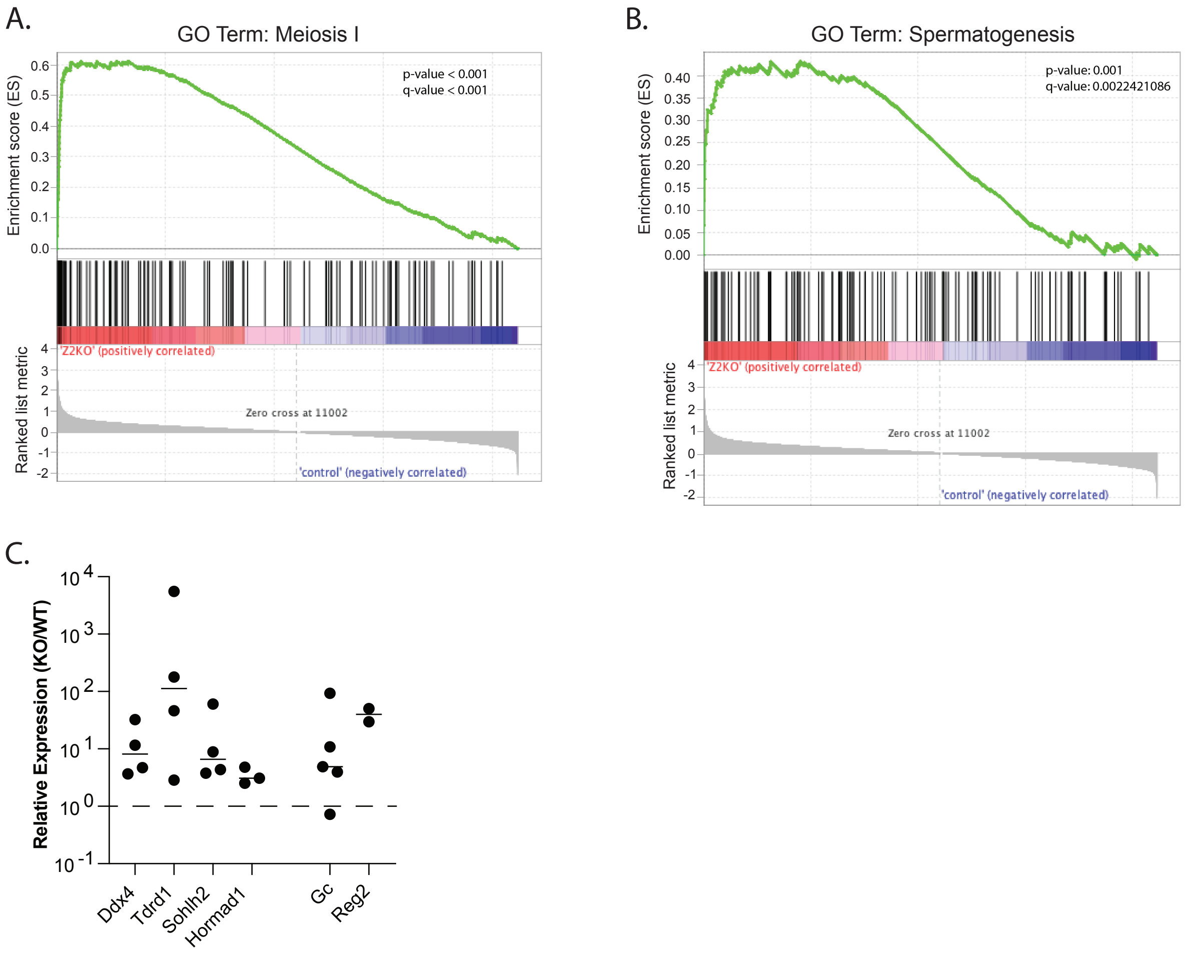

Supplementary Figure S2. Validation of upregulated targets in *Zmym2*$^{-/-}$ embryos. (A-B) GSEA analysis showing that genes involved in meiosis **I (A)** and spermatogenesis **(B)** show significantly higher expression in *Zmym2*$^{-/-}$ E8.5 embryos. **(C)** Quantitative reverse-transcription PCR (qRT-PCR) of four upregulated genes expressed from annotated promoter (*Ddx4*, *Tdrd1*, *Sohlh2*, *Hormad1*) and two LINE-fusions (*Gc*, *Reg2*). Each dot reflects the ratio of expression in KO/WT littermate embryos, with expression normalized to *Rps16*. The dashed line represents a 1:1 ratio in relative expression between WT and KO embryos.

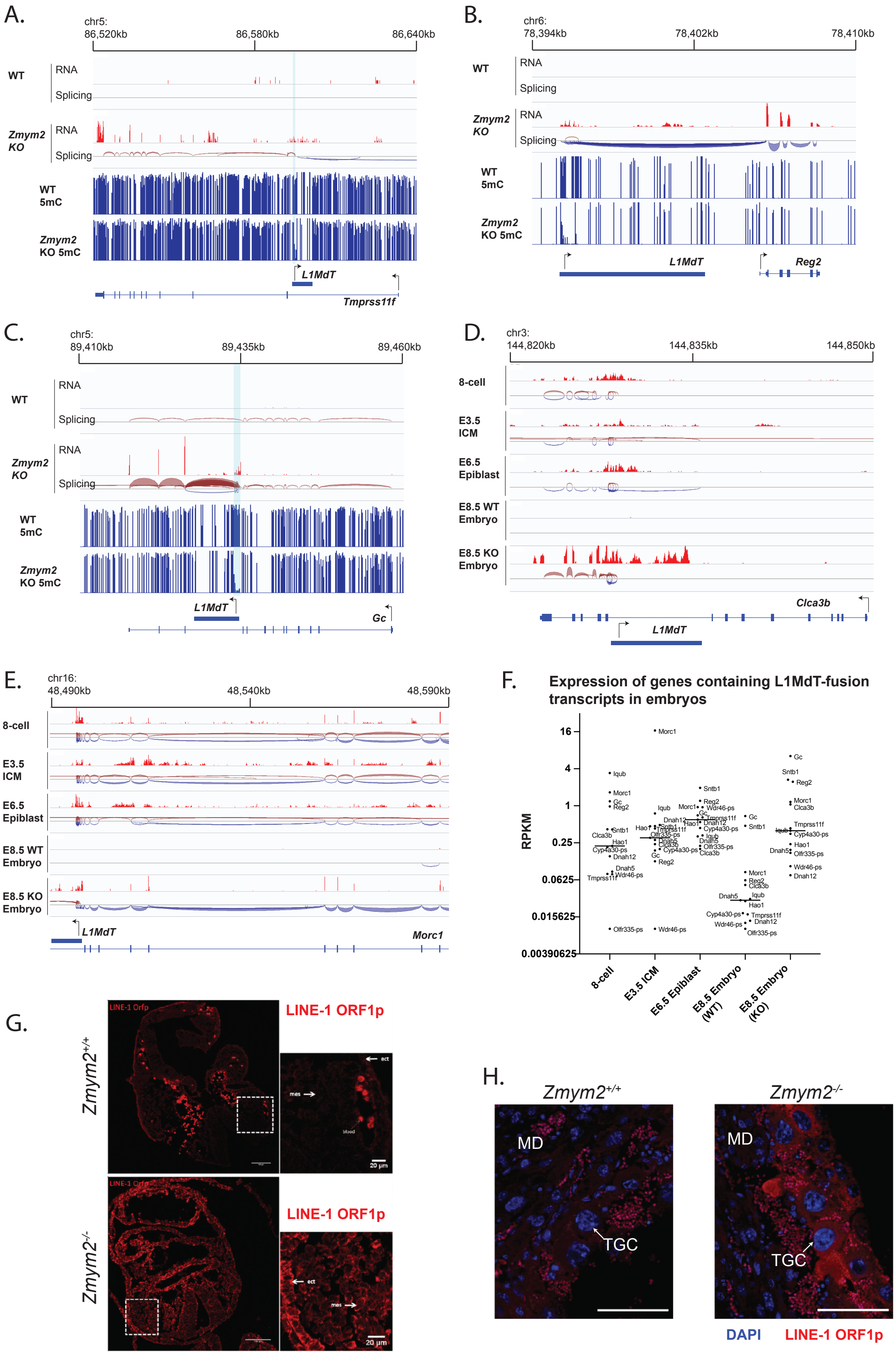

A.
chr5:
86,520kb
86,580kb
86,640kb
WT
RNA
Splicing
Zmym2 KO
WT 5mC
Zmym2 KO 5mC
L1MdT
Tmprss11f
B.
chr6:
78,394kb
78,402kb
78,410kb
Reg2
C.
chr5:
89,410kb
89,435kb
89,460kb
Gc
D.
chr3:
144,820kb
144,835kb
144,850kb
8-cell
E3.5 ICM
E6.5 Epiblast
E8.5 WT Embryo
E8.5 KO Embryo
Clca3b
E.
chr16:
48,490kb
48,540kb
48,590kb
Morc1
F.
Expression of genes containing L1MdT-fusion transcripts in embryos
RPKM
16
4
1
0.25
0.0625
0.015625
0.00390625
8-cell
E3.5 ICM
E6.5 Epiblast
E8.5 Embryo (WT)
E8.5 Embryo (KO)
G.
Zmym2+/+
Zmym2-/-
LINE-1 ORF1p
LINE-1 ORF1p
20 µm
H.
Zmym2+/+
Zmym2-/-
MD
TGC
DAPI
LINE-1 ORF1p

Supplementary Figure S3. Expression of L1MdT-gene fusion transcripts in *Zmym2$^{-/-}$* embryos and early embryonic development. (A-C) Representative examples of L1MdT-gene fusion transcripts. Splicing event coloured by direction of transcript (red =plus strand, blue= minus strand). Note examples of bidirectional transcription of an internal L1MdT element **(A)**, splicing from an upstream L1MdT in a forward direction into a gene **(B)** and splicing from an internal L1MdT promoter in a forward direction into a gene **(C)**. Hypomethylated DMRs are indicated in light blue. **(D, E)** Representative examples of L1MdT-gene fusion transcript expression in early embryonic development. In **D-E**, 8-cell, E3.5 and E6.5 RNA-seq data lack stranded-ness information and so direction of splicing is not informative. **(F)** Plot of genes containing L1MdT-gene fusion transcripts during early mouse embryonic development. Expression level of each gene is indicated. The horizontal line at each timepoint dictates the median RPKM. In **D-F**, 8-cell, E3.5 and E6.5 RNA-seq data are from Smith 2017. **(G)** Immunofluorescence staining of LINE-1 ORF1p (red) in E8.5 *Zmym2$^{+/+}$* and *Zmym2$^{-/-}$* embryos. Regions of mesoderm and ectoderm in inset are indicated. Scale bar= 20µm. **(H)** Immunofluorescence staining of LINE-1 ORF1p (red) and DAPI (blue) of E9.5 *Zmym2$^{+/+}$* and *Zmym2$^{-/-}$* implantation sites. TGC= trophoblast giant cell, MD= maternal decidua. Small red cells visible in both samples are maternal blood cells. Scale bars= 100µm.

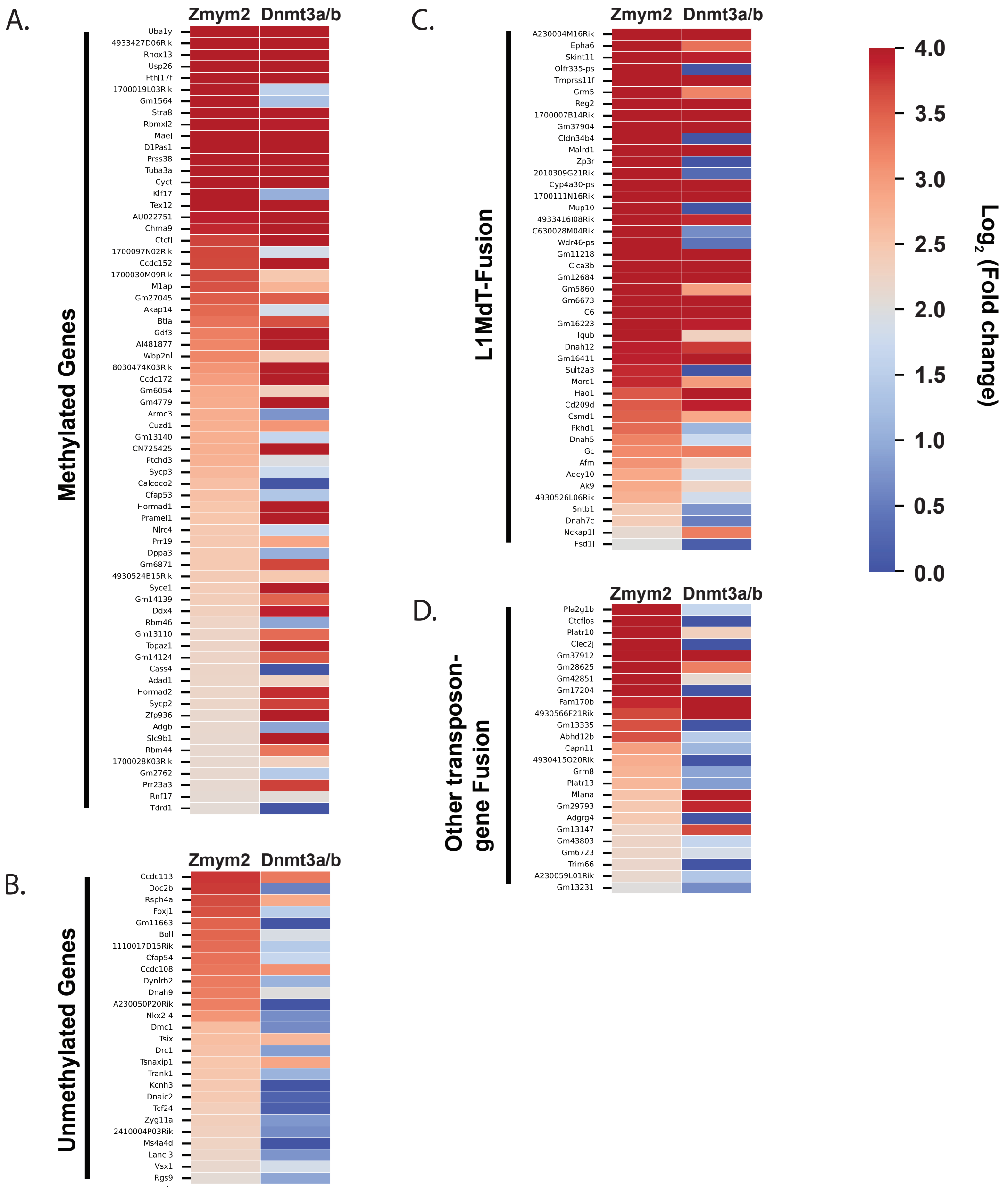

Supplementary Figure S4. Upregulation of ZMYM2 targets in *Dnmt3a/Dnmt3b*$^{-/-}$ mouse embryos. (**A-D**) Heatmap showing $\log_2$ fold change of each indicated upregulated gene in *Zmym2*$^{-/-}$ and *Dnmt3a/Dnmt3b*$^{-/-}$ E8.5 embryos relative to controls. (**A**) Genes transcribed from annotated TSS with methylated (>20% CpG methylation in 1kb around TSS) promoter. (**B**) Genes transcribed from annotated TSS with unmethylated (<20% CpG methylation in 1kb around TSS) promoter. (**C**) Genes transcribed from L1MdT promoter. (**D**) Genes transcribed from a transposon promoter other than L1MdT.

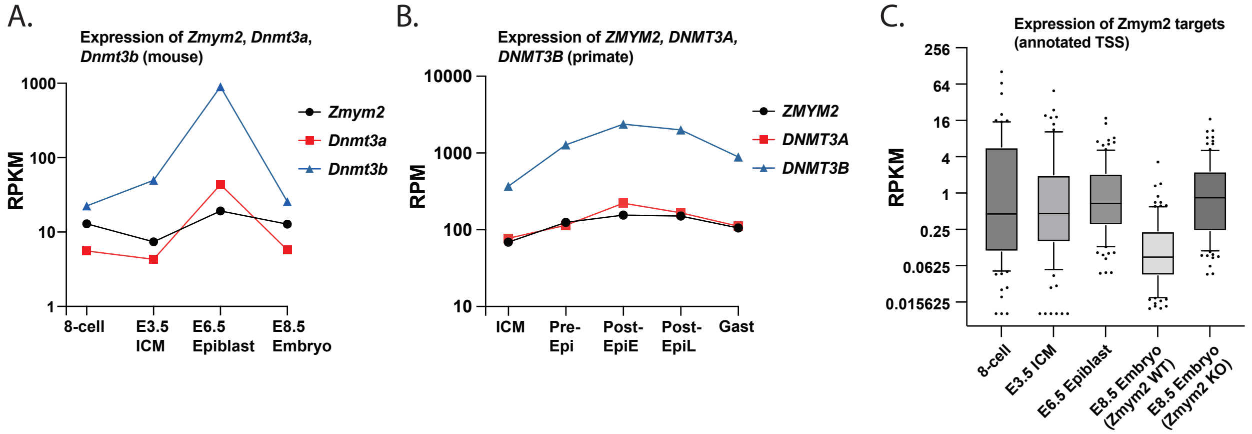

Supplementary Figure S5. Expression of ZMYM2, *de novo* methyltransferases, and ZMYM2 target genes during embryonic development.
(**A**) Expression of *Zmym2* and the *de novo* methyltransferases *Dnmt3a* and *Dnmt3b* during early murine embryonic development. Data from Smith 2017 (8 cell – E6.5) and this paper (E8.5). (**B**) Expression of *ZMYM2* and the *de novo* methyltransferases *DNMT3A* and *DNMT3B* during cynomolgus monkey development. Data from Nakamura 2016. (**C**) Expression of the 95 genes upregulated in *Zmym2*$^{-/-}$ and expressed from the annotated TSS during early murine embryonic development. Data from Smith 2017 (8 cell – E6.5) and this paper (E8.5).

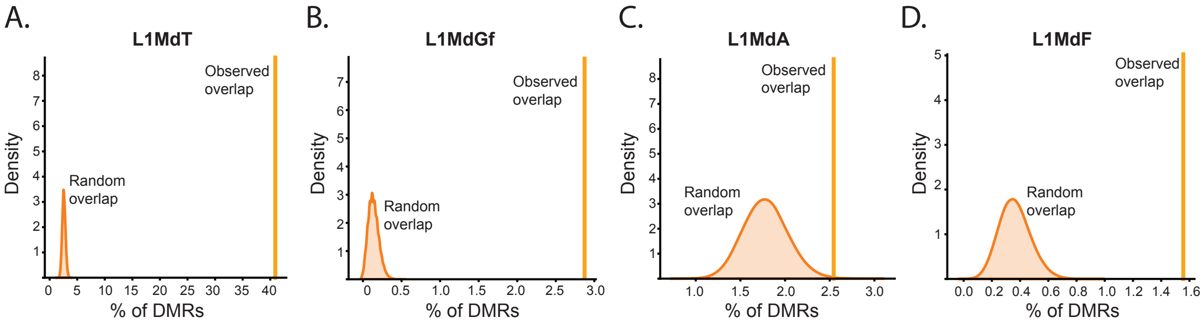

Supplementary Figure S6. Overlap of hypomethylated DMRs with L1Md elements.
(**A-D**) Monte-Carlo simulation was undertaken in which the positions of DMRs were randomly placed in the genome and repeated 100,000 times. The overlap with indicated transposon class is indicated as "Random overlap". This number is contrasted with the "observed overlap" number for actual DMRs. Results are shown for L1MdT (**A**), L1MdGf (**B**), L1MdA (**C**), L1MdF (**D**).

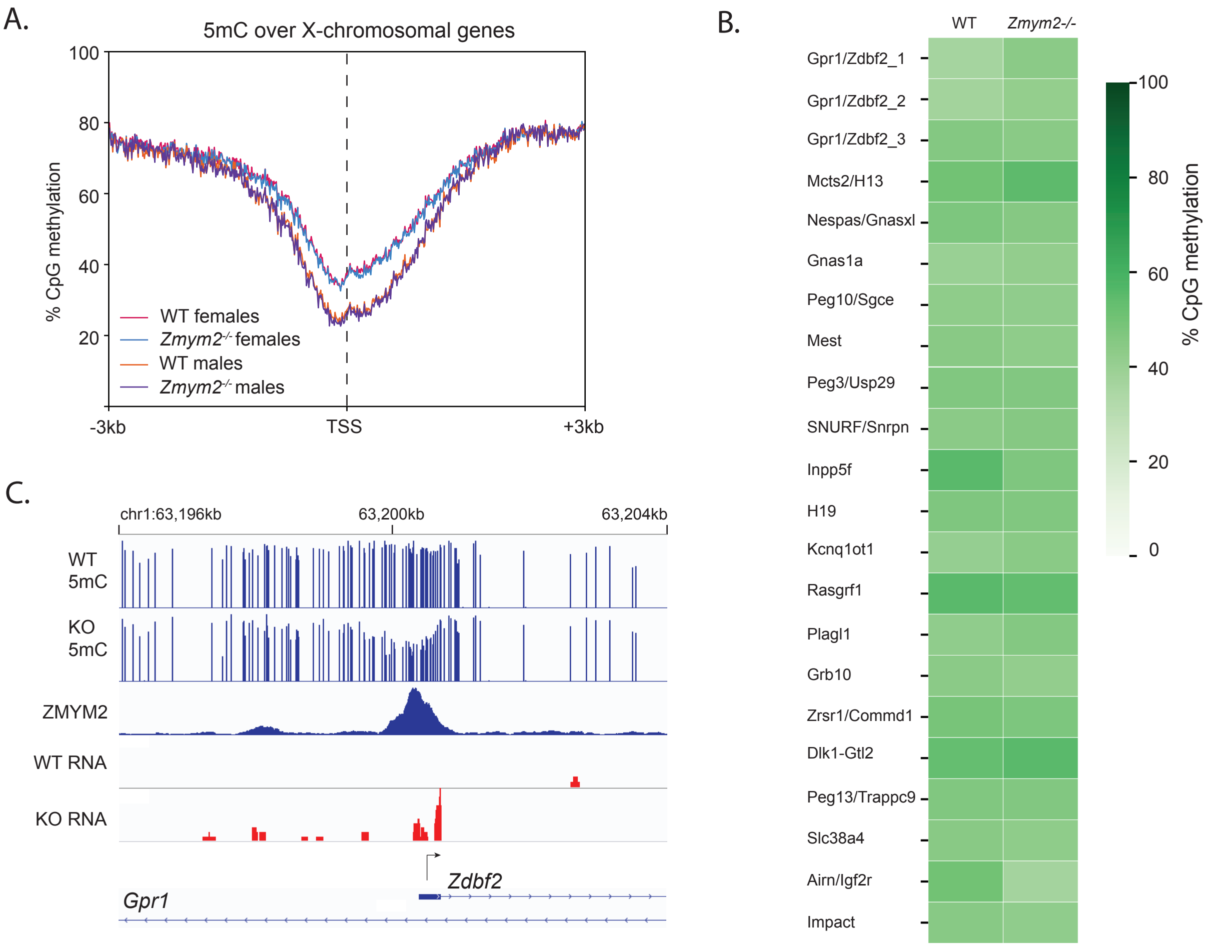

Supplementary Figure S7. Methylation of inactive X-chromosome and imprinted loci in *Zmym2*$^{-/-}$ embryos. (**A**) Metaplot of DNA methylation over X-chromosomal genes in *Zmym2*$^{+/+}$ and *Zmym2*$^{-/-}$ embryos. Note higher methylation in female than male, but no difference between *Zmym2*$^{+/+}$ and *Zmym2*$^{-/-}$. No statistically significant reduction in methylation is observed in *Zmym2*$^{-/-}$. (**B**) DNA methylation over murine stably imprinted regions in *Zmym2*$^{+/+}$ and *Zmym2*$^{-/-}$. No abnormalities are apparent. P-values were calculated from two-tailed unpaired T-test. Significance was determined after Bonferroni correction. Coordinates of imprinted loci are taken from Wang 2014. (**C**) Hypomethylation, and aberrant transcription from the *Liz/Zdbf2* transient imprint in *Zmym2*$^{-/-}$. Note enrichment of ZMYM2 at the target locus.

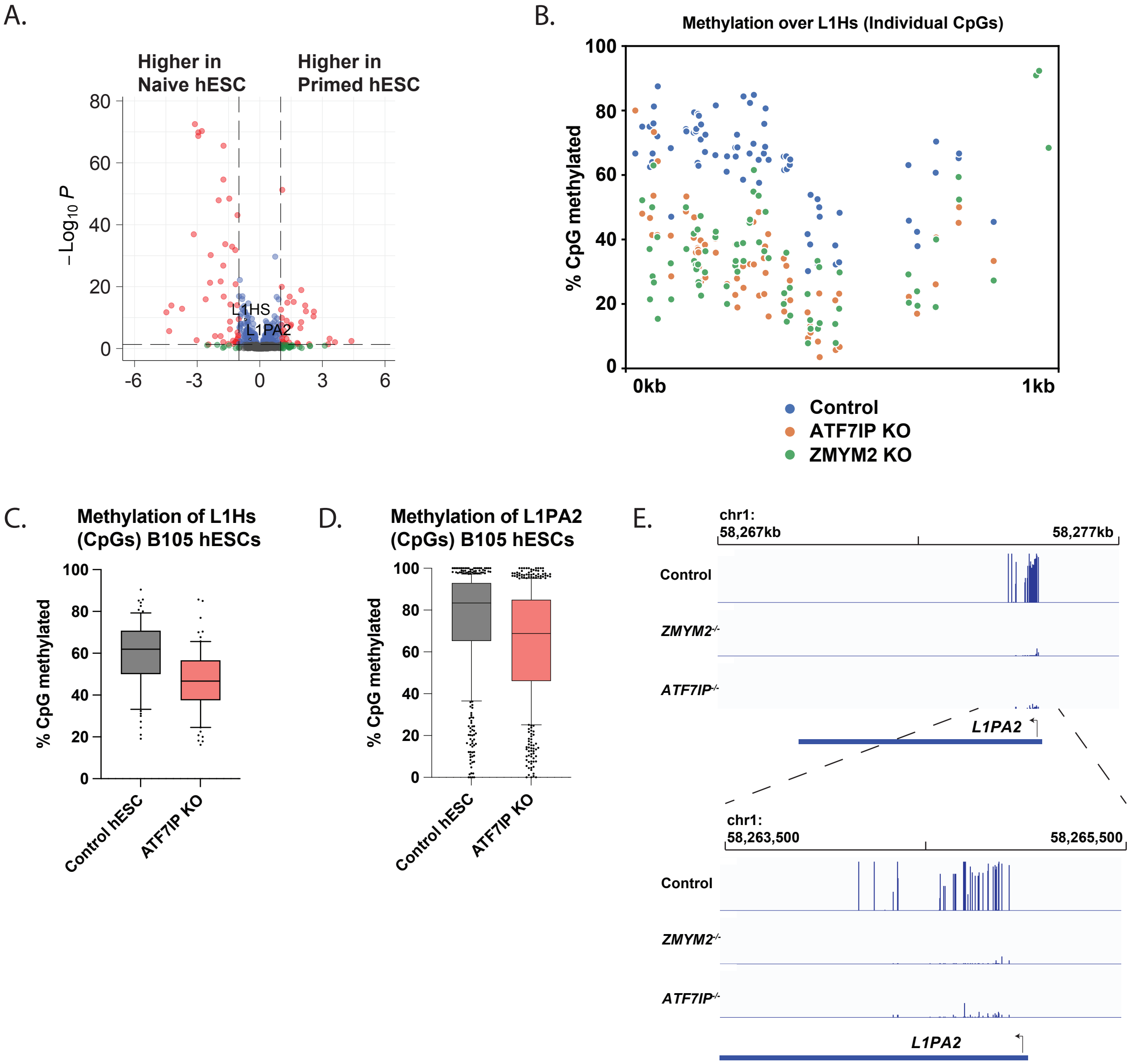

Supplementary Figure S8. Upregulation and hypomethylation of young LINE elements in *ZMYM2*$^{-/-}$ human embryonic stem cells.

(**A**) Relative expression of transposon families in naïve and primed hESCs. Note that naïve hESCs show only a very modest increase in L1Hs and L1PA2 levels. (**B**) Methylation levels of individual CpGs in an L1Hs consensus element for the genotype indicated. Only CpGs with >10-fold coverage in the Bar 2021 RRBS data are shown. (**C,D**) DNA methylation of L1Hs (**C**) and L1PA2 (**D**) control and *ATF7IP*$^{-/-}$ hESCs in B105 hESCs line. Note similarity to Figure 4B,C in this second hESC line. (**E**) Example of an L1PA2 element hypomethylated in *ZMYM2*$^{-/-}$ and *ATF7IP*$^{-/-}$ hESCs. Inset is shown beneath main figure.

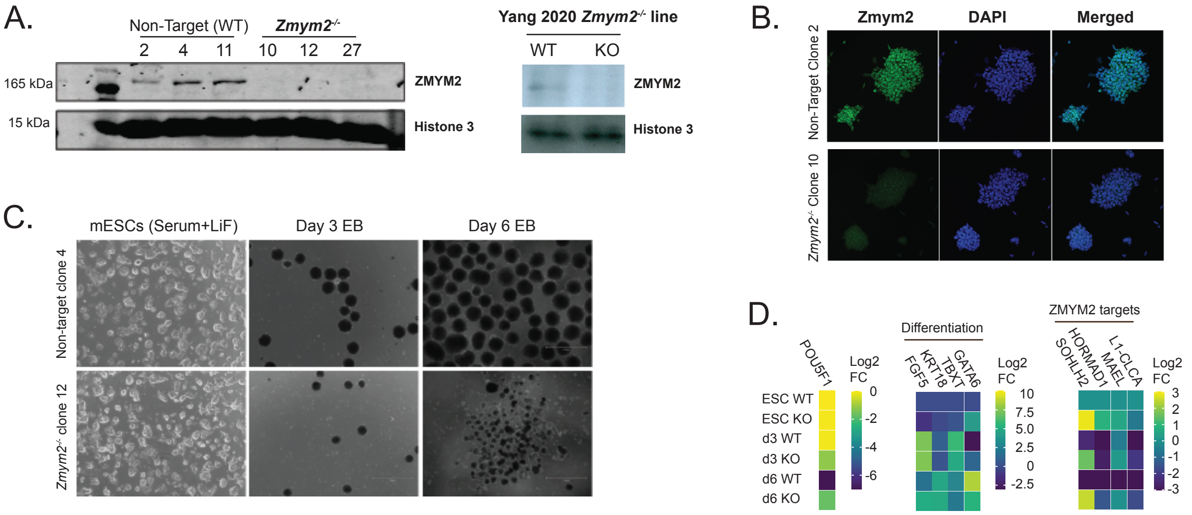

Supplementary Figure S9. Recapitulation of ZMYM2-mediated silencing in an Embryoid Body (EB) assay.

(**A**) Western blots of control and *Zmym2*$^{-/-}$ clonal mESC lines generated on a V6.5 background (left) as well as WT J1 and *Zmym2*$^{-/-}$ mESCs generated by Yang and colleagues. (**B**) Immunofluorescence staining of control and *Zmym2*$^{-/-}$ clonal line for ZMYM2. (**C**) Light microscopy images of *Zmym2*$^{+/+}$ and *Zmym2*$^{-/-}$ mESCs and embryoid bodies. Scale bar = 750 µm. (**D**) Relative expression of *Pou5f1*, differentiation genes, and ZMYM2-target genes in control mESCs and *Zmym2*$^{-/-}$ mESCs generated by Yang and colleagues, during embryoid body differentiation.

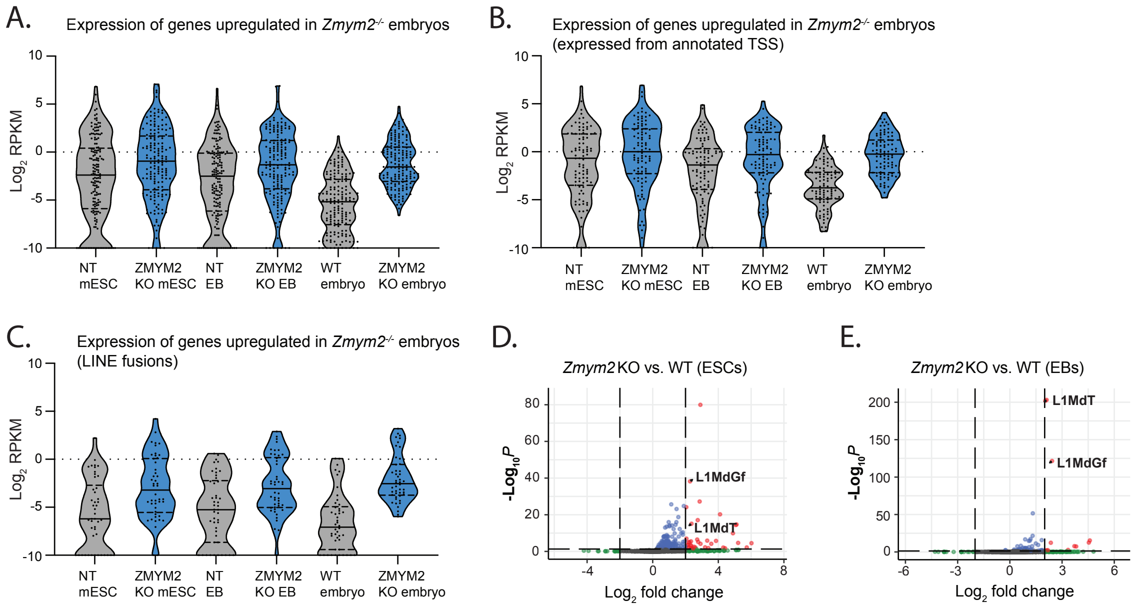

Supplementary Figure S10. Upregulation of ZMYM2 targets in *Zmym2$^{-/-}$* mESCs and EBs. (A) Violin plot showing expression of the 165 genes upregulated in *Zmym2$^{-/-}$* E8.5 embryos in the cell types indicated. Note elevated expression in KO rather than control (NT=Non-targeting sgRNA) **(B)** Violin plot showing expression of the 95 genes upregulated in *Zmym2$^{-/-}$* E8.5 embryos (expressed from TSS) in the cell types indicated. **(C)** Violin plot showing expression of the 46 LINE-fusion genes upregulated in *Zmym2$^{-/-}$* E8.5 embryos in the cell types indicated. **(D-E)** Volcano plot of differentially expressed transposable elements comparing *Zmym2$^{+/+}$* and *Zmym2$^{-/-}$* mESCs **(D)** and EBs **(E)**. Significant differentially expressed transposons (fold ≥ 4, q-value < 0.05) are coloured red.

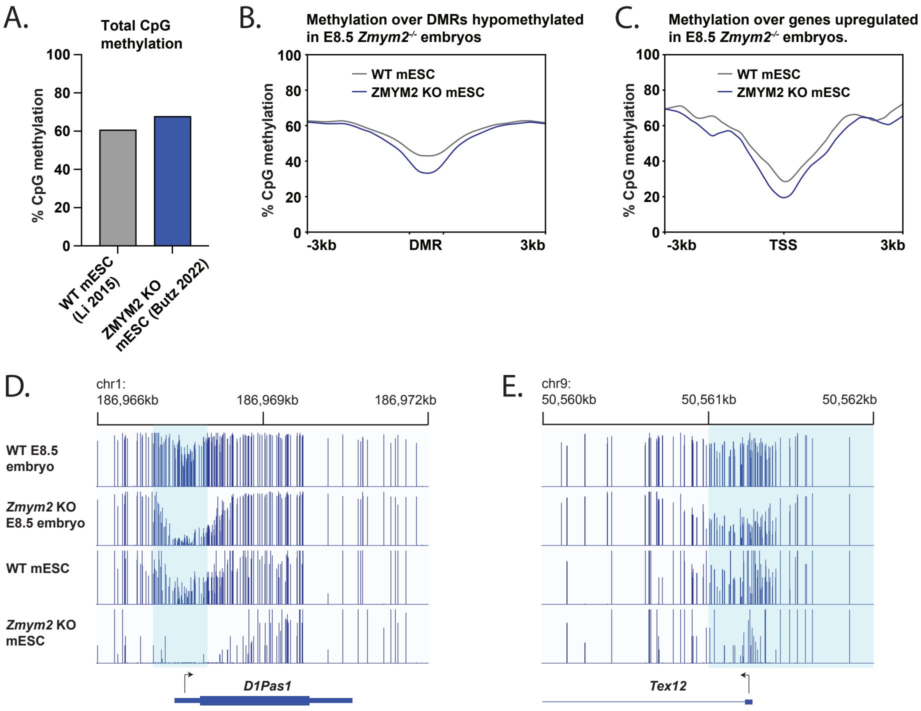

Supplementary Figure S11. Hypomethylation of ZMYM2 targets in *Zmym2$^{-/-}$* mESCs. (A) Global DNA methylation levels in published WT (Li 2015) and *Zmym2$^{-/-}$* mESCs (Butz 2022). **(B)** Metaplot of CpG methylation for WT and *Zmym2$^{-/-}$* mESCs over DMRs hypomethylated in *Zmym2$^{-/-}$* E8.5 mice. Note lower DNA methylation in *Zmym2$^{-/-}$* mESCs over DMRs despite higher genomewide methylation. **(C)** Metaplot of CpG methylation for WT and *Zmym2$^{-/-}$* mESCs over TSS of 95 upregulated genes in *Zmym2$^{-/-}$* embryos expressed from annotated TSS. **(D,E)** Examples of ZMYM2 targets with hypomethylated promoters in *Zmym2$^{-/-}$* mESCs.

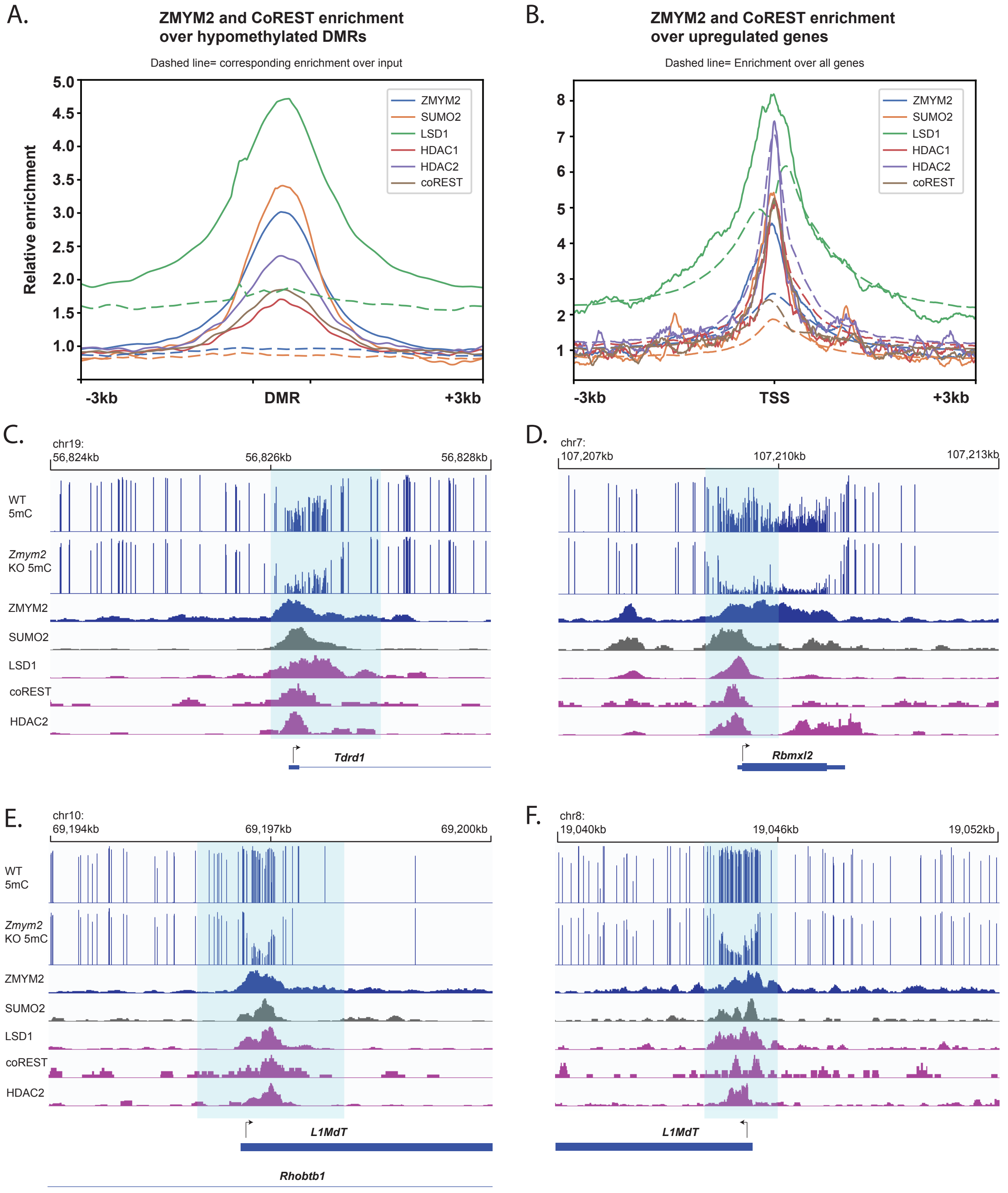

Supplementary Figure S12. Enrichment of ZMYM2 and CoREST components over regions hypomethylated in E8.5 *Zmym2*$^{-/-}$ embryos. (A-B) Enrichment of ZMYM2, SUMO2, and CoREST complex component ChIP-seq over regions hypomethylated in E8.5 *Zmym2*$^{-/-}$ embryos **(A)** and upregulated genes expressed from annotated TSS **(B)**. **(C-F)** ChIP-seq and DNA methylation over individual examples of upregulated genes **(C,D)** or LINE elements **(E,F)**. Hypomethylated DMRs are indicated with light blue boxes. All ChIP-seq data is published data from mESCs.For data in this figure, ChIP-seq is taken from Yang 2020 (ZMYM2, LSD1), Whyte 2012 (CoREST, HDAC1, HDAC2) and Yang 2015 (SUMO2).

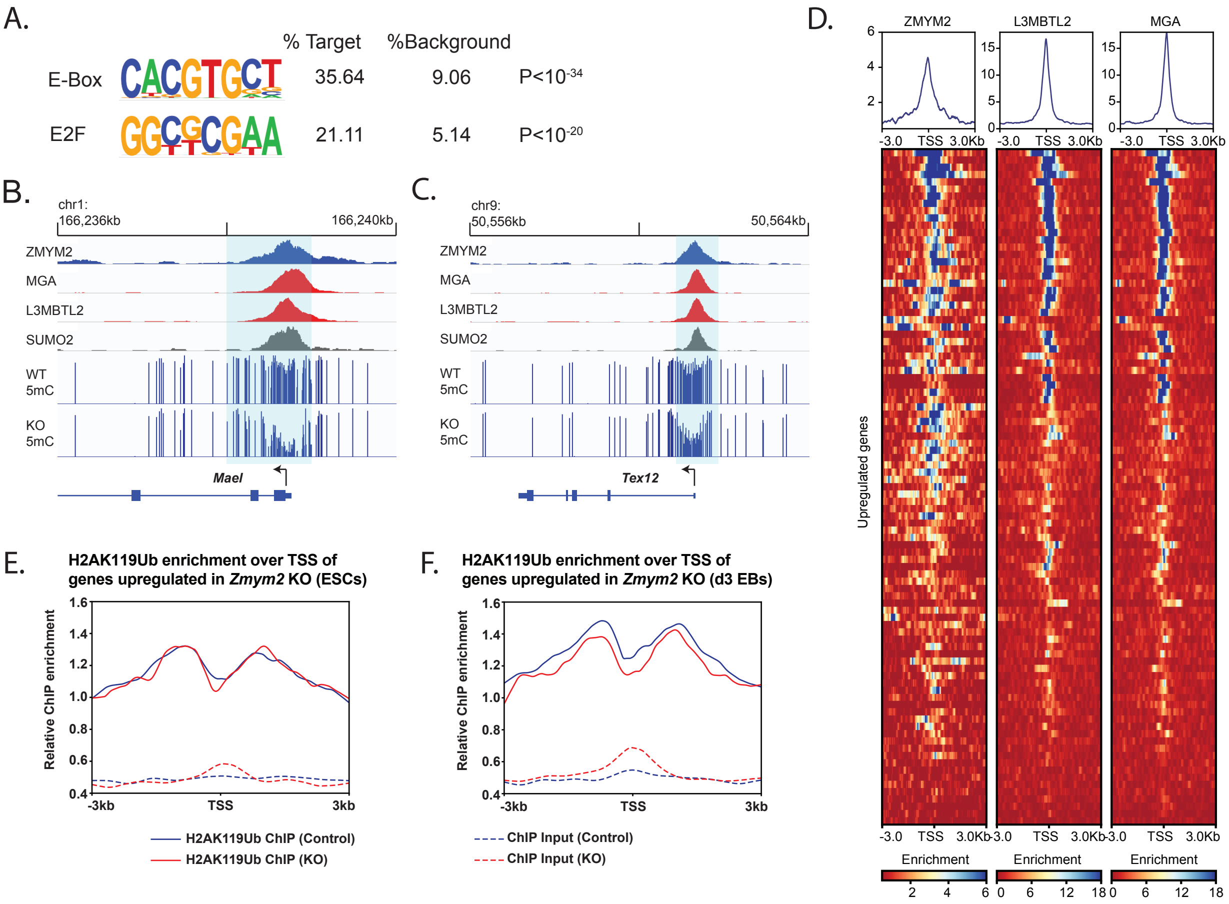

Supplementary Figure S13. ZMYM2 binding at PRC1.6 targets.

(**A**) HOMER motif analysis of Cluster 1 ZMYM2 peaks show enrichment of E-Box and E2F motifs relative to random (background) signal, consistent with targeting by the PRC1.6 components MGA and E2F6 respectively. (**B-C**) ChIP-seq and DNA methylation data over the *Mael* (**B**) and *Tex12* (**C**) loci. Note colocalization of ZMYM2 with PRC1.6 components and hypomethylated regions in *Zmym2*$^{-/-}$ E8.5 embryos. Hypomethylated DMRs are indicated with light blue boxes. (**D**) Heatmap of enrichment for ZMYM2 and the PRC1.6 components L3MBTL2 and MGA over the TSS of the ninety-five upregulated genes expressed from their annotated promoter.(**E,F**) Relative ChIP enrichment of H2AK119Ub in *Zmym2*$^{+/+}$ and *Zmym2*$^{-/-}$ mESCs (**E**) and day 3 EBs (**F**) over genes upregulated in *Zmym2*$^{-/-}$ (transposon-fusion genes excluded). Note that there is no difference between *Zmym2*$^{+/+}$ and *Zmym2*$^{-/-}$. The modestly increased input enrichment in *Zmym2*$^{-/-}$ may reflect increased locus accessibility as a result of impaired silencing.

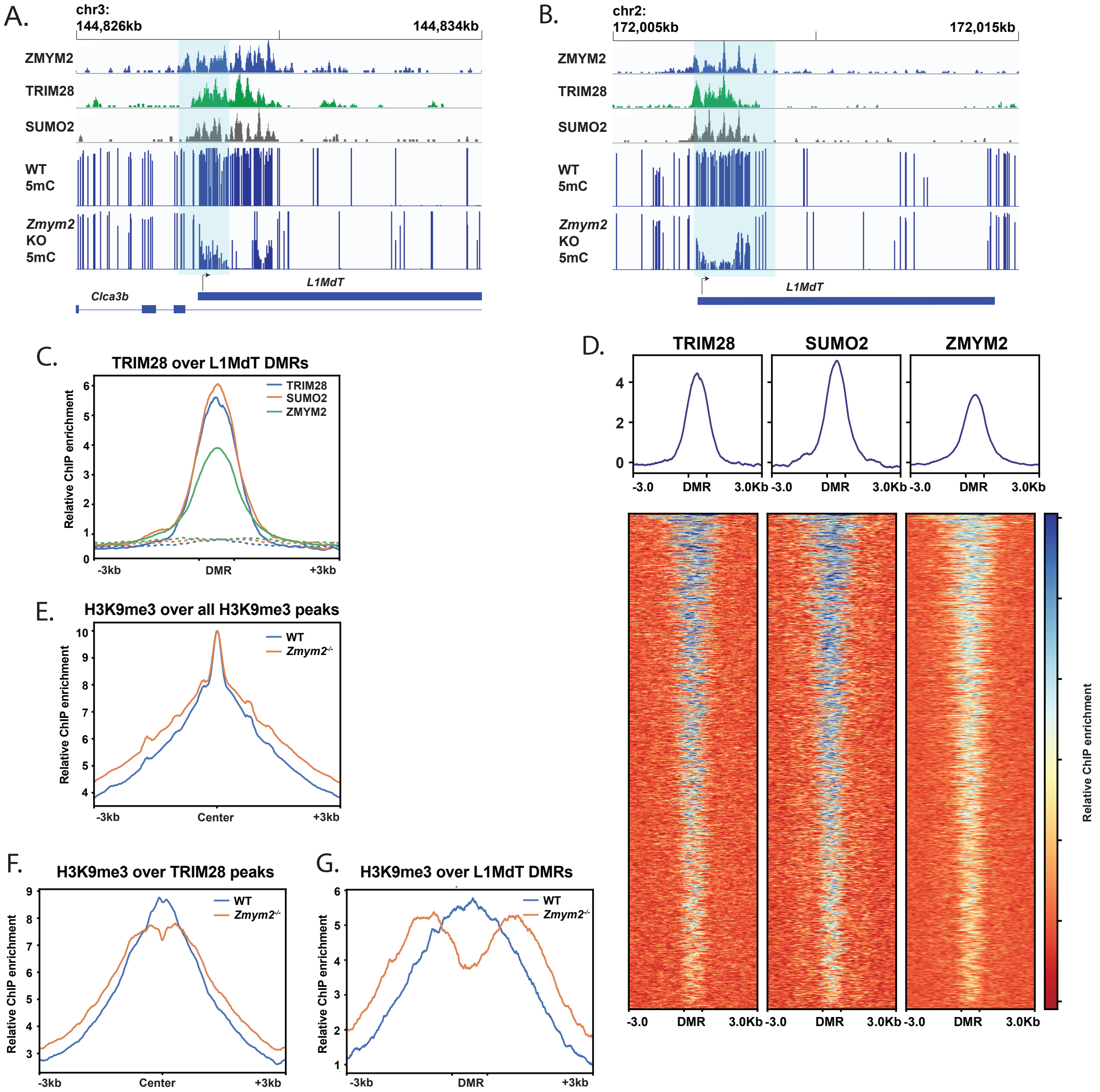

Supplementary Figure S14. ZMYM2 binding at TRIM28 targets.
(**A,B**) ZMYM2, TRIM28, SUMO2 ChIP-seq and WGBS data plotted over the *L1MdT-Clca3b* fusion transcript (**A**) and a representative LINE element (**B**). Note correspondence of the three proteins at L1MdT promoters and corresponding reduction in DNA methylation. (**C**) Metaplot of TRIM28, SUMO2 and ZMYM2 mESC ChIP-seq signal over DMRs hypomethylated in *Zmym2*$^{-/-}$ which overlap with L1MdT elements. Dashed lines indicate ChIP input. (**D**) Heatmap of input-normalized ZMYM2, TRIM28, SUMO2 ChIP-seq data over hypomethylated L1MdT DMRs. (**E**) Normalized H3K9me3 signal in control and *Zmym2*$^{-/-}$ mESCs (Butz 2022), metaplotted over a defined set of H3K9-methylated regions (Barrel 2022). (**F-G**) H3K9me3 in control and *Zmym2*$^{-/-}$ mESCs, metaplotted over TRIM28 peaks (**F**) and L1MdT DMRs (**G**).

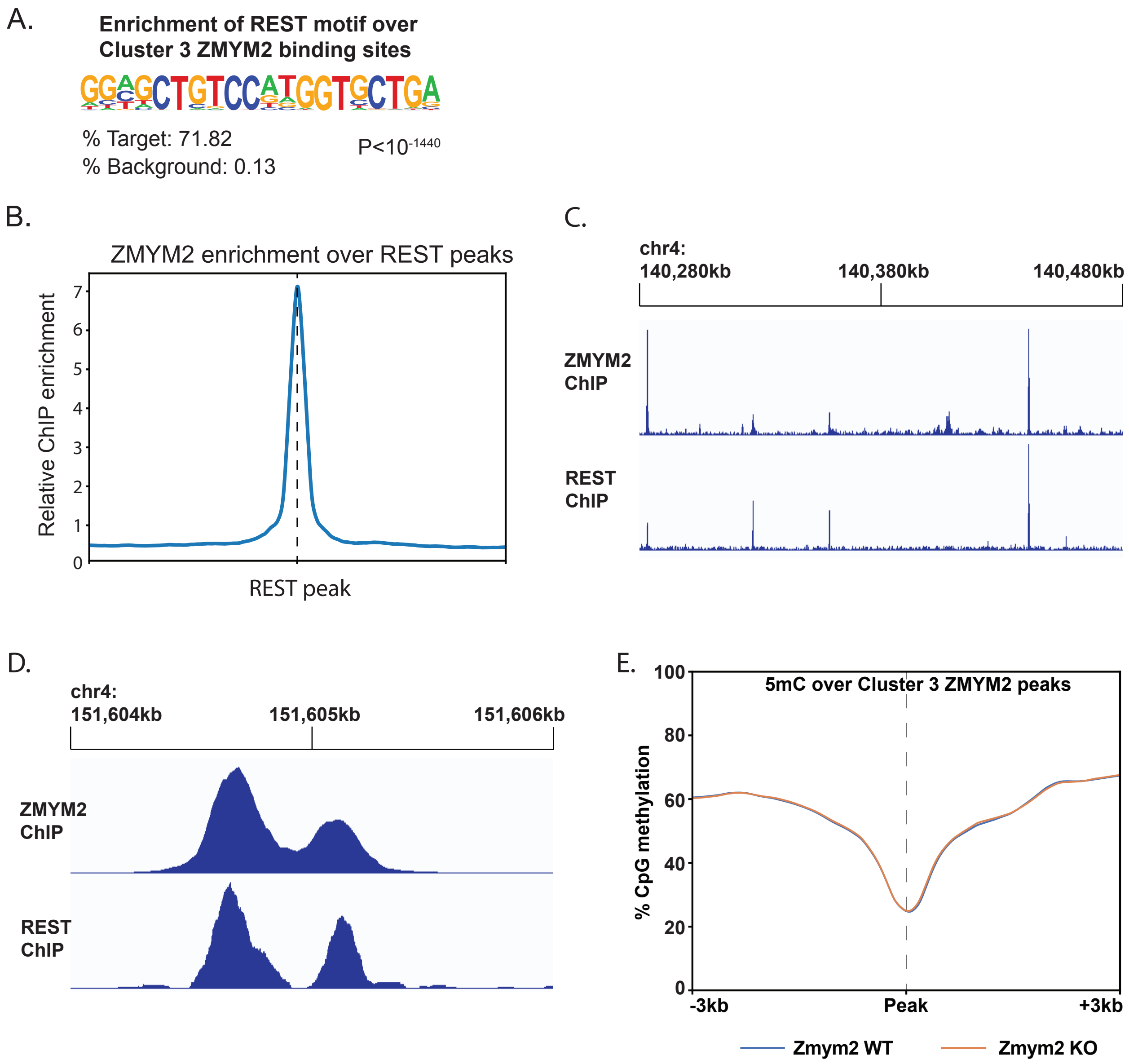

Supplementary Figure S15. ZMYM2 binding at REST targets.
(**A**) HOMER motif analysis of Cluster 3 ZMYM2 peaks show enrichment of the REST binding site. (**B**) ZMYM2 ChIP-seq enrichment over REST binding sites. (**C,D**) Overlap of REST and ZMYM2 ChIP-seq peaks over a stretch of chromosome 4. (**E**) DNA methylation over Cluster 3 (REST-enriched) peaks in *Zmym2*$^{+/+}$ and *Zmym2*$^{-/-}$ embryos.

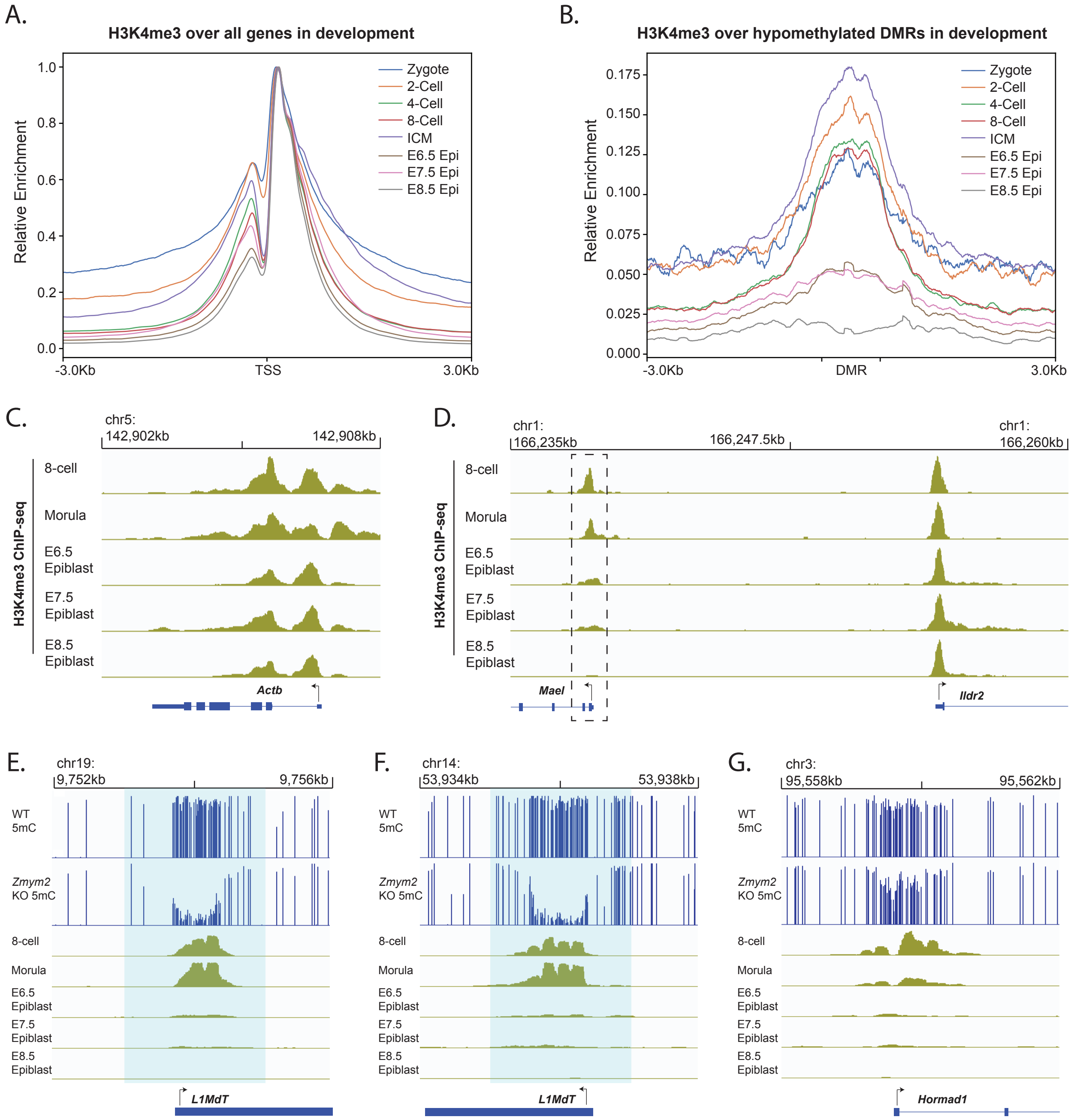

Supplementary Figure S16. H3K4 methylation at ZMYM2-target sites throughout development. **(A)** Metaplot of H3K4 methylation over all genes after normalization across indicated embryonic stages. (**B**) H3K4me3 over regions hypomethylated in *Zmym2*$^{-/-}$ embryos across cell types during embryonic development. Note that these regions naturally have elevated levels of H3K4 methylation from the 2-cell through the ICM stage, then lose H3K4me3 methylation post-implantation. (**C**) H3K4me3 enrichment over housekeeping gene *Actb*. (**D**) Normalized H3K4me3 over *Mael* promoter in cell types indicated. An unaffected locus (*Ildr2*) is shown for comparison. (**E,F**) Examples of LINE elements with hypomethylated promoters in *Zmym2*$^{-/-}$ embryos that show a dramatic reduction in H3K4me3 during the course of normal development. (**G**) H3K4me3 over the promoter of *Hormad1*, a gene upregulated in *Zmym2*$^{-/-}$ embryos.

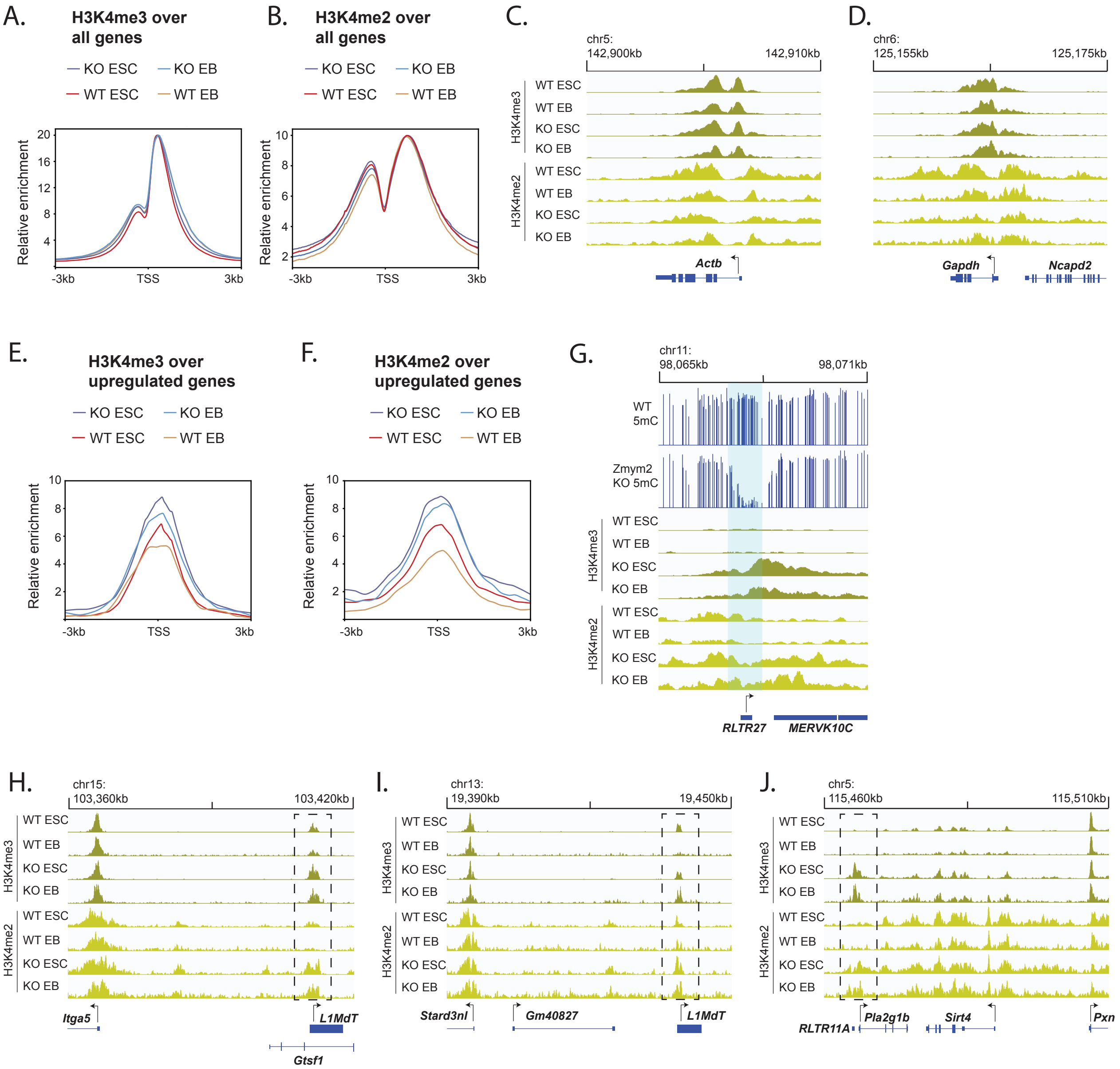

Supplementary Figure S17. Aberrant H3K4 hypermethylation at ZMYM2-target sites in *Zmym2*$^{-/-}$ mESCs. (**A, B**) Metaplot of H3K4me3 (**A**) and H3K4me2 (**B**) over all genes after normalization in indicated samples. Note similar pattern of enrichment over all replicates, including narrow H3K4me3 and broader H3K4me2 enrichment. (**C, D**) H3K4me3 and H3K4me2 ChIP enrichment over housekeeping *Actb* and *Gapdh* genes. (**E, F**) H3K4me3 and H3K4me2 ChIP-seq enrichment over genes upregulated in *Zmym2*$^{-/-}$ embryos. (**G**) A striking example of increased H3K4me2/3 over a hypomethylated DMR, an RLTR27 transposon in this case, in *Zmym2*$^{-/-}$ mESCs and EBs. (**H-J**) Examples of H3K4 hypermethylation in *Zmym2*$^{-/-}$ cells.
