# Supplementary material for: ZMYM2 is essential for methylation of germline genes and active transposons in embryonic development
Source: Nucleic Acids Res. 2023 Jul 3;51(14):7314–29. doi: 10.1093/nar/gkad540 (PMC10415128; doi:10.1093/nar/gkad540)
Supplement: gkad540_Supplemental_Files [file gkad540_supplemental_files.zip › Supplementary Table captions.docx]

**SUPPLEMENTARY TABLE LEGENDS**

**Supplementary Table S1. Mapping statistics for next generation sequencing data generated or used in the course of this project.**

**Supplementary Table S2. RPKMs of all genes in control and *Zmym2^-/-^* embryos, mESCs, and EBs.**

**Supplementary Table S3. Sequences of primers used in this project.**

**Supplementary Table S4. Annotations of upregulated genes in *Zmym2^-/-^* embryos.**

**Supplementary Table S5. Locations of DMRs in *Zmym2^-/-^* embryos.**
